# The Sulphate of Cinchonidia

**Published:** 1876-10

**Authors:** Robert Battey

**Affiliations:** Rome, Ga.


VOL. XIV.] OCTOBER—1876. [No. 7

### Original Communications.

### THE SULPHATE OF CINCHONIDIA.

BY ROBERT BATTEY, M.D., ROME, GA.

In the process of manufacture of the sulphate of quinine from the various species of cinchona bark, several other alkaloids are found to be associated with quinine in varying proportions. These alkaloids—cinchonia, cinchonidia, quinidia, etc.—are isolated as by-products, and are offered by the manufacturers at prices so low as to render the question of their antiperiodic power one of especial interest to country practitioners of medicine who dispense their own remedies. Nearly twenty years ago I instituted a series of trials to test the relative antiperiodic power of cinchonia as compared with quinine, and made a publication embodying the results; since which time I have employed the cinchonia and its sulphate in my practice; not exclusively, but much more largely than quinia. I am now fully satisfied that in the employment of cinchonia in the place of quinia, to the extent I have carried it, my patients have been gainers and not losers. The result of these years of trial has but served to confirm me in the good opinion I had formed of the cinchonia alkaloid.

Much more recently the attention of the profession has been called to the other alkaloids, and especially to cinchonidia; and during five months past, with occasional exceptions, both my copartner (Dr. Holmes) and myself have used the cinchonidia to the exclusion of both quinia and cinchonia. The object of the present writing is to bring before the profession the results of our conjoined observation in the use of this remedy.

First, as to its pharmaceutical qualities. The color, texture, form of crystal, and taste of cinchonidia sulphate are much more closely allied to the quinia salt than to the corresponding salt of cinchonia; indeed, the cinchonidia would scarcely be distinguished by the patient from quinia, even when dispensed in powder. Like quinia, it is readily dispensed in solution by the addition of a minute proportion of acid; but the solution has not the iridescent appearance of the quinia solution. It is worked into pill with the same facility as quinia, with either crumb of bread, honey, or elixir vitriol. In taste its bitterness is somewhat less intense than quinia.

I have long been in the habit of dissolving the cinchonia sulphate in acidulated water, and precipitating the alkaloid by ammonia. The precipitate is washed and dried, forming a pure white powder, much like calcined magnesia, which has but little taste when taken into the mouth, until time enough elapses to effect its very slow solution in the saliva. The powder I combine with one-tenth its weight of pulverized liquorice root, and administer it to children in lieu of quinine. Its bitter taste is so slight as not to be objected to, and its therapeutical effects I find to be entirely satisfactory. When the cinchonidia sulphate is treated in the same manner, a similar white powder results, and the combination is not to be distinguished from the other by the sense of taste, excepting that it is slightly more soluble, and probably a little more active as a febrifuge, but less eligible for the preparation of a "tasteless quinine" for children. The combination I find dissolves readily in the acidulous stomach of a child, and offers so many advantages over the quinia sulphate that it really seems an unnecessary cruelty to administer the latter salt to children.

As a febrifuge and antiperiodic, we have administered the cinchonidia to a large number of cases. We are not able to perceive any difference in remedial power as compared with quinia. It is more inclined to nauseate a delicate stomach than quinia, but not so much so as the cinchonia. In this respect, however, we have not found the cinchonidia so objectionable as to interfere materially with its use, excepting when exhibited in very large doses. Any nauseating qualities of either cinchonia or cinchonidia, in the form of sulphate, may be corrected in a large degree by exhibiting either remedy in the alkaloid form, with powdered liquorice, as before indicated for children. The cinchonidia very rarely produces *tinnitus aurium*, though sometimes it will do so. In general it may be advantageously used when quinine is especially objectionable in this respect, and when cinchonia, which we find entirely free from such effects, is not well borne by the stomach.

We are in the habit, in certain cases, of exhibiting quinia in large doses, administering from thirty to sixty grains within a few consecutive hours, for its antipyretic and antiphlogistic effects, securing a condition of nervous and vascular action which we are accustomed to designate by the term "quininism." This state we find to follow equally upon the use of the cinchonidia, and with no appreciable differences between the two, excepting when the stomach is inclined to be irritable, when the full dose of quinine is better borne than the cinchonidia.

We also use the alkaloids of cinchona bark combined with the muriated tincture of iron and simple syrup, for tonic purposes. In this form we are not able to perceive any appreciable difference between quinia, cinchonidia, and cinchonia, and hence give the preference to the last, as being much the cheapest in price.

In cases where we have not felt entirely willing to rely upon the cinchonidia as an antiperiodic in pernicious intermittents, after the use of quinine for the arrest of the paroxysms, we have employed the former agent subsequently as a prophylactic, with results entirely satisfactory.

In conclusion, after a somewhat extended experience with the cinchonidia, we are of opinion that its therapeutical value is positive and unequivocal; we shall continue its use in the future, and esteem it a valuable addition to our armamentarium.

